# Beyond formal equivalence: research questions and explanatory function in group and multilevel selection theory

**DOI:** 10.1007/s12064-026-00495-6

**Published:** 2026-10-07

**Authors:** Pedro Henrique Martins, Fausto Nomura

**Affiliations:** Graduate Program in Ecology and Evolution, Institute of Biological Sciences, Universidade Federal de Goiás, Goiânia, Goiás, Brazil

**Keywords:** Group selection, Multilevel selection, Inclusive fitness, Price equation, Social evolution, Evolutionary theory

## Abstract

The debate over group and multilevel selection remains central to evolutionary biology, particularly in explanations of cooperation and social evolution. A persistent issue is whether group- and multilevel-selection models and kin-selection formulations are formally equivalent, that is, whether they can represent the same evolutionary change under specified assumptions, and what follows from such equivalence for explanation. We conducted a systematic review and thematic synthesis of 94 model-centered studies, characterized by explanatory orientation, Research Question, multilevel selection I/II classification, formal tool, decomposition role, causal interpretation, and equivalence claims. Explanatory orientations were heterogeneous, and formal-tool use overlapped substantially across them. Associations involving decomposition role weakened in sensitivity analyses, while predominant Research Question showed no detectable association with multilevel selection I/II classification. Among the 68 studies that substantively discussed equivalence, qualified positions predominated and categorical rejection was rare; most addressed multiple objects of equivalence. These results indicate that explanatory orientation, Research Questions, formal representations, causal interpretation, and equivalence claims are related but non-interchangeable dimensions of the debate. Formal equivalence can establish genuine relations between representations without determining that they identify the same causal structure, serve the same explanatory function, or support the same attribution of adaptation. Persistent disagreement is therefore better understood as concerning what formal translations preserve, which biological questions they answer, and what explanatory conclusions follow from them.

**Supplementary Information:**

The online version contains supplementary material available at https://doi.org/10.1007/s12064-026-00495-6.

## Introduction

The evolution of cooperation and altruism remains a central problem in evolutionary biology. In its standard biological sense, altruism refers to behaviour that imposes a fitness cost on the actor while increasing the fitness of another individual. Selfish behaviour, by contrast, increases the actor’s fitness without producing a corresponding benefit to the recipient (Kerr et al. 2004; West et al. 2006). This creates a classical explanatory difficulty: if natural selection operates through differential reproductive success, behaviours that reduce the actor’s fitness while benefiting others should, at first sight, be eliminated by individual-level selection (Cronin 1999; Kay et al. 2019). Yet costly cooperative behaviours are widespread in nature, including reproductive altruism in eusocial insects, alarm calls in vertebrates, cooperative breeding, and other forms of social behaviour in which individual costs and social benefits appear to be unequally distributed (Dugatkin 2002; Foster 2008; West et al. 2006; West and Gardner 2010).

One of the earliest explanations for this problem was group selection (Darwin 1859, 1871; Wynne-Edwards 1962, 1963). In its classical form, group selection proposed that, although selfish individuals may outperform altruists within groups, groups composed of altruistic individuals may outperform groups composed mainly of selfish individuals (Wilson 1980). Under this interpretation, cooperative traits could evolve because groups with more altruists would be more productive, more persistent, or less likely to go extinct than groups dominated by selfish individuals (Wilson 1980). Before the consolidation of the critique of group selection in the 1960s, explanations in terms of group benefit or species benefit were relatively common in evolutionary reasoning, especially in discussions of social behaviour, population regulation, and the apparent functionality of traits at levels above the individual (Okasha 2001, 2006).

This explanatory tradition changed substantially after the critiques of Williams (1966) and Maynard Smith (1964). Williams argued that traits interpreted as group adaptations should not be explained by selection at the group level when the same phenomena could be accounted for more parsimoniously by selection acting on individuals. Maynard Smith’s analysis of early group-selection models, including the haystack model, reinforced the view that group selection would require restrictive conditions, such as small groups, low migration, high relatedness within groups, and differential extinction or reproduction of whole groups (Boyd and Richerson 1990; Crow and Aoki 1982; Eshel 1972; Leigh 2010; Maynard Smith 1964; Williams 1966). Without such conditions, selfish individuals would tend to invade altruistic groups, and within-group selection would usually be stronger than between-group selection. As a result, group selection came to be viewed by many evolutionary biologists as an implausible, weak, or unnecessary explanation for cooperation (Maynard Smith 1964; Williams 1966; Okasha 2001, 2006).

During the same period, alternative explanations for cooperation became dominant. Kin selection explained how social behaviour could evolve when actors increase their inclusive fitness by helping relatives who carry copies of the same genes (Hamilton 1964a, b). Reciprocal altruism showed how cooperation could be favoured through repeated interactions among individuals (Trivers 1971). Evolutionary game theory provided formal models of strategic interaction and evolutionary stability (Maynard Smith and Price 1973). Together with gene-centred interpretations of natural selection, these approaches contributed to the decline of group-selection explanations and to the consolidation of individual- and gene-centred frameworks for the study of social evolution (Dawkins 1976; Borrello 2005; West et al. 2008).

However, the rejection of classical group selection did not end the controversy. From the 1970s and 1980s onward, new theoretical, experimental, and philosophical work reopened the debate about whether selection can act simultaneously at different levels of biological organization (Wilson 1975, 1983; Wade 1977, 1978; Wade and McCauley 1980; Sober and Wilson 1998; Borrello 2005; Okasha 2006). This renewed debate did not simply restore the older claim that traits evolve “for the good of the group”. Instead, it reframed the issue in terms of group-structured populations, trait groups, contextual effects, and alternative ways of partitioning evolutionary change, including within-group and between-group components. In this context, multilevel selection became less a return to classical group selection than a broader framework for analysing how group structure and social context affect the direction, strength, and interpretation of selection.

A central point in this renewed controversy concerns the relation between group selection, multilevel selection, kin selection, and inclusive fitness. The Price equation played an important role in this debate because it provides a general formalism for partitioning evolutionary change into components associated with covariance between trait and fitness and with changes transmitted across generations (Price 1970, 1972; Frank 2012). When applied to group-structured populations, a multilevel Price partition can express evolutionary change in between-group and within-group terms. These terms, however, do not determine the causal levels at which selection acts by themselves. In some causal structures, a between-group component can appropriately represent higher-level selection; in others, individual-level selection may also contribute to fitness differences between groups, so that only part of the between-group difference corresponds to higher-level selection (Jeler 2020; Shelton and Michod 2020). In such cases, contextual analysis may provide a more appropriate representation of the causal structure (Okasha 2016; Jeler 2020). The causal interpretation of a partition therefore depends on the process generating the observed associations, rather than on the algebraic form alone. Accordingly, a Price partition can be causally informative when its terms and assumptions appropriately represent the causal structure of the process under study. For some authors, the formal translatability of alternative partitions supports the view that group selection and kin selection are equivalent descriptions under specified conditions, since both can express the conditions under which cooperation evolves in group-structured populations (Queller 1992; Lehmann et al. 2007; Gardner et al. 2011; Marshall 2011; Lehtonen 2020). For others, formal equivalence does not eliminate important differences between approaches, because alternative models may identify different causal structures, depending on different assumptions, or answer different biological questions (Sober and Wilson 1998; Okasha 2006, 2016; Birch and Okasha 2015; van Veelen 2005, 2020; Jeler 2020; Lehtonen 2020).

Part of this disagreement derives from ambiguity in the expression “unit of selection”. Following Lloyd’s framework, what has often been treated as a single question can be separated into distinct Research Questions (Lloyd 1994, 2024; Suárez and Lloyd 2023). The interactor Research Question asks which entity interacts with the environment in such a way that variation in its traits is associated with differential success. The replicator/reproducer Research Question asks which entity transmits the relevant heritable structure across generations. The manifestor-of-adaptation Research Question asks at which level adaptations should be attributed, especially in the stronger sense of engineering adaptation, rather than merely as a selection-product adaptation. The beneficiary Research Question asks who benefits from the process of evolution by selection, either in the sense of possessing adaptations or in the sense of being the ultimate beneficiary over evolutionary time (Lloyd 1994, 2024; Suárez and Lloyd 2023). These questions can point to different levels of organization in the same evolutionary scenario. Accordingly, identifying selection or an interactor at a given level does not by itself establish engineering adaptation at that level. Thus, disagreement about group selection may persist not because authors simply use different terms, but because they use similar terms while answering different Research Questions.

A second source of ambiguity concerns the distinction between multilevel selection I (MSI) and multilevel selection II (MSII). In MSI, the focal outcome is the change in frequency of individual traits in a group-structured population; groups function as social or causal contexts that affect individual fitness. In MSII, the focal outcome is the change in frequency of groups or collectives themselves, which requires groups to differ in persistence, reproduction, fission, extinction, or colonization. The distinction has been influential in separating these two ways of representing multilevel selection, although its explanatory significance has also been questioned. Gardner (2015), for example, shows that genetical formulations of multilevel selection do not map straightforwardly onto the classical MSI/MSII distinction, while Bourrat (2023) questions whether MSI and MSII should always be interpreted as distinct evolutionary processes. The distinction nevertheless remains useful for identifying differences in what is changing, how fitness is attributed, and the relation between group structure and evolutionary change (Damuth and Heisler 1988; Mayo and Gilinsky 1987; Okasha 2006; Lloyd 2024).

These conceptual distinctions are connected to two broader explanatory traditions in multilevel evolutionary theory. The Evolutionary Change tradition, associated with the Wrightian tradition, is primarily concerned with the dynamics and causal organization of evolutionary change in group-structured populations. Its focus includes evolutionary change, interactors, and the effects of group structure and social context on selection, without ordinarily requiring that an interactor at a given level also be a manifestor-of-adaptation at that same level. By contrast, the Adaptationist/KS tradition, associated with Fisherian, kin-selection, and inclusive-fitness approaches, is primarily concerned with optimality, evolutionary stability, and the explanation of engineering adaptations. In this tradition, the interactor and manifestor-of-adaptation Research Questions are often coordinated, because the aim is not only to identify a level at which selection acts, but also to explain how a functional adaptation at that level could evolve and be maintained (Goodnight and Stevens 1997; Goodnight 2013; Lloyd 2024; Suárez and Lloyd 2023). These orientations describe differences in explanatory emphasis rather than exclusive methodological commitments: formal tools such as Price-equation partitions, contextual analysis, inclusive-fitness formulations, quantitative-genetic models, optimization, or game theory can be used in different explanatory contexts and therefore do not, by themselves, determine a study’s orientation.

The contemporary controversy over group and multilevel selection is therefore not exhausted by the question of whether alternative models are formally equivalent. Formal equivalence may show that two models can be translated into one another under particular assumptions, but it does not by itself establish predictive, causal, or explanatory equivalence, nor does it establish that the models address the same Research Question (Okasha 2016; Jeler 2020; Lehtonen 2020; Thies and Watson 2021). Models may be mathematically comparable while serving different functions: accounting for total evolutionary change, diagnosing components of selection, identifying causal relations, or explaining engineering adaptation. Conversely, differences in formal representation do not necessarily imply differences in the underlying evolutionary process. The explanatory significance of formal translation therefore depends on what is being represented, which assumptions connect the formalism to the causal structure of the system, and what the model is intended to explain. Clarifying these distinctions is necessary for understanding why the debate remains active despite decades of formal analysis.

In this review, we examine how the model-centered studies retrieved by our search organize the debate over group and multilevel selection. Drawing on a systematic review and thematic synthesis of 94 studies, we characterize the reviewed corpus according to predominant explanatory orientation, predominant Research Question, MSI/MSII classification, predominant formal tool, primary decomposition role, and position on equivalence. We examine whether explanatory orientation is associated with formal-tool use, decomposition role, and equivalence position, and whether predominant Research Question is associated with the MSI/MSII classification. We argue that the controversy is structured less by a simple opposition between “group selection” and “individual selection” than by differences in Research Questions, explanatory functions, and causal commitments. The central question is therefore not only whether formal equivalence holds in particular formulations, but what follows from that equivalence for causal interpretation and explanatory function.

## Materials and methods

### Search strategy and study selection

We conducted a systematic literature search and thematic synthesis to identify studies concerned with the formulation, comparison, or critical assessment of models of group selection, multilevel selection, kin selection, inclusive fitness, and altruism in the evolution of social behaviour. The review followed principles of thematic synthesis, qualitative research synthesis, and structured coding (Thomas and Harden 2008; Sandelowski et al. 2006; Saldaña 2016). The search was conducted on the Web of Science and Scopus on 4 July 2025, with no additional update search conducted thereafter. The search was designed to capture both explicitly multilevel models and studies that framed social evolution in terms of altruism, group selection, or model-based explanations.

The search was applied to title and keyword fields using the following Boolean structure: (“multilevel selection” OR “group selection” OR “altruism” OR “altruistic behavio*”) AND “model*”. The asterisk was used as a truncation operator, allowing “behavio*” to capture spelling variants and derived forms such as “behavior”, “behaviour”, “behavioral”, and “behavioural”. The full database-specific search strings are provided in Online Resource 1. The initial search retrieved 174 records from Web of Science and 419 records from Scopus. Records were imported into R environment (R Core Team 2021), merged using the bibliometrix package (Aria and Cuccurullo 2017), and duplicates were removed, resulting in 441 unique records after the removal of 152 duplicates.

**Fig. 1:**
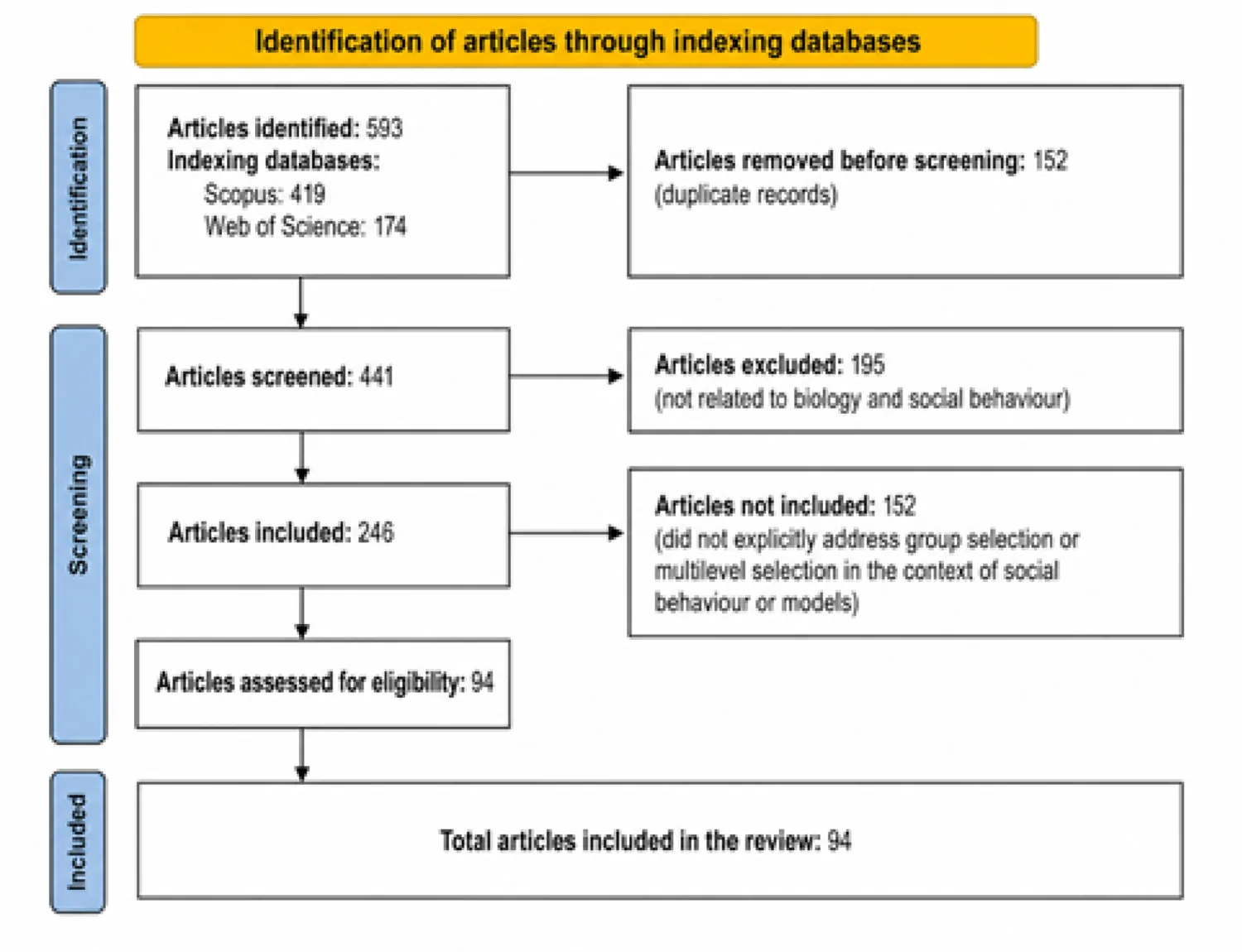
Study-selection flow diagram

Title and abstract screening were conducted using the *abstract screener* function from the metagear package (Lajeunesse 2016). Screening proceeded in two stages. First, records were retained when they were related to biology and social behaviour, resulting in 246 studies. Records were excluded at this stage when they referred to non-biological uses of group selection or altruism, did not address social behaviour or social traits, or were unrelated to evolutionary modelling. In the second stage, we retained only studies that explicitly addressed group selection or multilevel selection in the context of social behaviour, social traits, altruism, or genetic models of social evolution. Studies were excluded when they did not engage with group selection or multilevel selection, or did not contribute to the formulation, comparison, or critical assessment of models relevant to the review question. The final dataset comprised 94 studies included in the thematic synthesis. The study-selection process is summarized in Fig. 1.

The search retrieved 593 records from Scopus and Web of Science. After duplicate removal and two screening stages, 94 studies were included in the final thematic synthesis.

### Thematic synthesis and coding

The 94 studies were organized into a coding matrix following a thematic synthesis and structured coding approach (Thomas and Harden 2008; Sandelowski et al. 2006; Saldaña 2016). Each study received a unique numerical identifier and was coded for bibliographic, conceptual, and formal-model attributes. Bibliographic fields included title, year of publication, and authors; conceptual fields included the study’s stated objective, primary explanandum, main conclusion, adaptive explanatory target, evolutionary-change target, predominant explanatory orientation, predominant and secondary Research Questions, MSI/MSII classification, and position on equivalence; and formal and analytical fields included predominant formal tool, use and primary role of decomposition, and the causal status attributed to decomposition. Additional descriptive notes, including main findings and more fine-grained model descriptions, were retained in the supplementary coding matrix for traceability but were not used as variables in the contingency-table analyses.

Coding combined thematic synthesis with structured categorical classification: the thematic component reconstructed how each study framed the relation between group selection, kin selection, multilevel selection, causality, and explanatory function, whereas the categorical component summarized recurrent patterns across the reviewed corpus through contingency tables. All coding decisions were made by the first author. Online Resource 1 provides the coding matrix, coding rules, variable dictionary, analytical decisions, evidence locations, confidence assessments, and fine-grained entries used in the analyses, while the main coding dimensions are summarized in Table 1.

**Table 1:** Coding dimensions used in the thematic synthesis

Coding dimension	Main categories	Analytical use
Predominant explanatory orientation	Adaptationist/KS; Evolutionary Change; Hybrid; Unclassifiable/other	Characterizes the principal explanatory orientation of each study and provides the grouping variable for C1, C2, and C4
Adaptive explanatory target	Central; Secondary; Absent; Unclear	Records the explanatory importance assigned to functional or engineering adaptation, optimality, fitness maximization, or adaptive stability and contributes to the classification of predominant explanatory orientation
Evolutionary-change target	Central; Secondary; Absent; Unclear	Records the explanatory importance assigned to evolutionary dynamics, trait-frequency change, contextual effects, interactors, or the causal organization of evolutionary change and contributes to the classification of predominant explanatory orientation
Predominant research question	RQ1 interactor; RQ2 replicator/reproducer; RQ3 manifestor-of-adaptation; RQ4 beneficiary; Composite; NA	Identifies the principal evolutionary role or question addressed by each study. Directly comparable RQ classifications are used in C3
MSI/MSII classification	MSI; MSII; Mixed; NA	Characterizes whether the focal multilevel process concerns groups primarily as contexts for individual evolutionary change, groups as units undergoing differential persistence or reproduction, or a combination of both; used in C3
Predominant formal tool	Decompositional/statistical; Optimization/games; Dynamic/stochastic; Mixed/multiple; Other/undefined	Identifies the formal approach carrying the main inferential weight of each study; used in C1
Primary decomposition role	Accounting/diagnostic; Causal-explanatory; Methodological/comparative; Mixed; Not used	Characterizes the principal role assigned to decomposition; used in C2
Causal interpretation of decomposition	Causally agnostic; Causally interpreted; Conditional/underdetermined; Causal interpretation rejected; Not applicable/unclear	Records how the causal status of a decomposition is interpreted independently of its primary role
Equivalence object	Formal/mathematical; Predictive/empirical; Causal/explanatory; Multiple; Not applicable/unclear	Identifies the sense or combination of senses in which equivalence is discussed; used in the descriptive synthesis of equivalence
Detailed equivalence position	Equivalent/formally equivalent without explanatory qualification; Formally equivalent, explanatorily distinct; Mixed/case-dependent; Not equivalent; Not discussed/NA; Unclear	Characterizes the substantive position adopted by each study. Substantive positions are reported descriptively and are collapsed only for the exploratory C4 contingency analysis

RQ = Research Question; MSI = multilevel selection I; MSII = multilevel selection II; NA = not applicable or not directly classifiable. Composite denotes studies in which no single predominant RQ could be retained for direct comparison in C3. Detailed coding rules, auxiliary fields, confidence assessments, and the complete coding matrix are provided in Online Resource 1

To characterize broad explanatory orientations in the reviewed corpus, the distinction between the Adaptationist/KS and Evolutionary Change traditions was used as a conceptual heuristic (Goodnight and Stevens 1997; Goodnight 2013; Lloyd 2024; Suárez and Lloyd 2023). The unit of classification was the predominant explanatory orientation of each study, guided by the question: “What is the principal explanatory payoff claimed by the study?” Evidence was evaluated from the explicitly stated objective or research question, primary explanandum, main interpretation, principal conclusion, and the roles assigned to adaptation and evolutionary change. Author identity and classifications assigned to other publications by the same author were not used as classification criteria.

Two explanatory targets were evaluated separately. The adaptive explanatory target concerned whether a study primarily sought to explain functional or engineering adaptation, optimality, fitness maximization, evolutionary stability as an adaptive outcome, or the conditions under which a functional trait is favoured or maintained. The evolutionary-change target concerned whether a study primarily sought to explain changes in trait frequencies or population states, the dynamics of selection, contextual effects, interactors, or the causal organization of evolutionary change. Both targets were coded as Central, Secondary, Absent, or Unclear. For the adaptive explanatory target, trait increase, invasion, or persistence alone was insufficient for a Central classification unless favourability, stability, or functional significance constituted a principal explanatory endpoint. For the evolutionary-change target, dynamics were classified as Secondary when they served primarily to derive conditions of favourability, stability, or adaptive maintenance rather than constituting an explanatory endpoint in their own right.

Predominant explanatory orientation was then coded as Adaptationist/KS, Evolutionary Change, Hybrid, or Unclassifiable/other. Adaptationist/KS was assigned when adaptive explanation constituted the principal explanatory payoff and evolutionary change was secondary or instrumental; Evolutionary Change was assigned when evolutionary dynamics or causal organization constituted the principal payoff and adaptive explanation was secondary or absent. Hybrid was assigned when both explanatory targets were central and neither was clearly subordinate. Unclassifiable/other was assigned when no predominant orientation could be identified, the principal explanandum fell outside both orientations, or the study primarily evaluated the scope, validity, or proper use of a formal method without making either explanatory target central. Classification considered the stated objective, primary explanandum, main interpretation, and conclusion, but excluded independent analytical dimensions such as formal tool, decomposition role, equivalence position, and MSI/MSII classification. Accordingly, the presence of the Price equation, contextual analysis, game theory, optimization, inclusive fitness, population genetics, quantitative genetics, multilevel modelling, or within- and between-group decompositions did not by itself determine explanatory orientation.

Each study was also coded according to the predominant Research Question addressed in Lloyd’s framework (Lloyd 1994, 2024; Suárez and Lloyd 2023). RQ1, the interactor Research Question, was assigned when the study primarily asked where selection acts or which entity interacts with the environment in a way that generates differential success. RQ2, the replicator/reproducer Research Question, was assigned when the focus was on the entity responsible for transmitting the relevant heritable structure across generations. RQ3, the manifestor-of-adaptation Research Question, was assigned when the study primarily concerned the level at which engineering adaptation, optimization, or adaptation in the stronger functional sense should be attributed. RQ4, the beneficiary Research Question, was assigned when the study primarily asked which entity benefits from the process of evolution by selection. Secondary Research Questions were recorded when another RQ played a relevant role but was not the focus of the study. Studies without a directly comparable predominant RQ were retained in the coding matrix but excluded from the RQ × MSI/MSII contingency analysis.

The MSI/MSII classification followed the distinction between multilevel selection I and multilevel selection II (Damuth and Heisler 1988; Mayo and Gilinsky 1987; Okasha 2006; Lloyd 2024). The classification was coded as multilevel selection I (MSI), multilevel selection II (MSII), Mixed, or NA. MSI was assigned when the group functioned mainly as a context affecting the fitness or evolutionary success of individual traits. MSII was assigned when groups, collectives, colonies, demes, or other higher-level entities were treated as units undergoing differential persistence, reproduction, fission, extinction, or colonization. Mixed was used when a study explicitly combined or moved between MSI and MSII logic. NA was retained when the study did not allow a clear classification.

Drawing on discussions of formal equivalence and its limits in the social-evolution literature (Okasha 2016; Jeler 2020; Lehtonen 2020; Thies and Watson 2021), equivalence was coded according to whether it was substantively discussed, the object of the equivalence claim, and the position adopted by the study. Equivalence discussion was first recorded as Yes or No. Among studies with a substantive discussion, the object of equivalence was classified as Formal/mathematical, Predictive/empirical, Causal/explanatory, or Multiple, while the detailed position was classified as Equivalent/formally equivalent without explanatory qualification, formally equivalent, explanatorily distinct, Mixed/case-dependent, or Not equivalent. The first category included studies that affirmed equivalence or formal translatability without an explicit material restriction on its explanatory significance; the second was reserved for studies that explicitly accepted formal equivalence while denying or restricting explanatory equivalence. Studies without a substantive discussion were coded as Not applicable/unclear for equivalence object and Not discussed/NA for detailed position. The proportion of studies discussing equivalence and the distributions of equivalence objects and detailed positions were used as the primary descriptive characterization of this dimension. Detailed cross-tabulations of position by explanatory orientation and by equivalence object were also examined descriptively.

For the contingency analysis relating explanatory orientation to collapsed equivalence position, the substantive detailed positions were collapsed into Affirms equivalence and Restricts/rejects equivalence. Affirms equivalence included studies whose predominant conclusion affirmed equivalence or formal translatability without a material explanatory restriction. Restricts/rejects equivalence combined formally equivalent, explanatorily distinct, Mixed/case-dependent, and Not equivalent positions. This collapsed variable provided a secondary exploratory summary of the general direction of equivalence positions and did not replace the detailed position or equivalence object in the descriptive synthesis.

The predominant formal tool was classified according to the tool carrying the main inferential weight of the study, rather than the presence of any particular formalism. Categories were Decompositional/statistical, Optimization/games, Dynamic/stochastic, Mixed/multiple, and Other/undefined. Mixed/multiple was used when two or more tools were co-primary and no single predominant tool could be identified.

Decomposition was coded separately from formal tool. Studies were first classified according to whether they substantively used, derived, interpreted, or evaluated a decomposition of evolutionary change, selection, fitness, covariance, variance, or effects across levels. When decomposition was used, its primary role was classified as Accounting/diagnostic, Causal-explanatory, Methodological/comparative, or Mixed; otherwise it was classified as not used. Accounting/diagnostic referred to uses that primarily partitioned, quantified, translated, or diagnosed components. Causal-explanatory referred to uses in which decomposition contributed centrally to identifying causal pathways, levels, mechanisms, or causal contributions. Methodological/comparative referred to uses whose main purpose was to compare, unify, evaluate, or criticize alternative methods, partitions, assumptions, or domains of validity. Mixed was used when two or more roles were co-primary.

The causal interpretation of decomposition was recorded separately as causally agnostic, causally interpreted, Conditional/underdetermined, Causal interpretation rejected, or not applicable/unclear. Causally agnostic indicated that the decomposition was treated as an identity or statistical partition without an intrinsic causal interpretation; Causally interpreted indicated that the study assigned the decomposition a causal interpretation in the focal system; and Conditional/underdetermined indicated that causal adequacy depended materially on assumptions about the generating process, group definition, omitted pathways, intervention, or information beyond the algebraic identity and observed associations. Causal interpretation rejected was used when the focal causal interpretation was explicitly rejected.

The quantitative synthesis comprised four planned contingency analyses: C1, predominant explanatory orientation × predominant formal tool; C2, predominant explanatory orientation × primary decomposition role; C3, predominant Research Question × MSI/MSII classification; and C4, predominant explanatory orientation × collapsed equivalence position. Two sensitivity analyses evaluated the influence of the Unclassifiable/other category on C2: C2-S1 excluded this category and included 78 studies, whereas C2-S2 retained only Adaptationist/KS and Evolutionary Change studies and included 60. C1 and C2 included all 94 studies, C3 included the 81 studies with both a directly comparable predominant RQ and a substantive MSI/MSII classification, and C4 included the 68 studies with a substantive equivalence position. Studies in which equivalence was not substantively discussed were retained in the coding matrix but excluded from C4.

Observed counts and row percentages were used as the primary descriptive outputs. Row percentages were used because the number of studies differed among categories. Pearson’s chi-square statistics and expected counts were calculated following standard methods for contingency-table analysis (Agresti 2018). Adjusted standardized residuals were examined following Haberman (1973), Monte Carlo *p*-values were used to accommodate sparse contingency tables (Hope 1968), and bias-corrected Cramér’s V was calculated as an effect-size diagnostic (Bergsma 2013). Because several tables contained sparse expected counts, Monte Carlo p-values were estimated using 10,000 simulations with seed = 123. Cells with absolute adjusted standardized residuals ≥ 1.96 were treated as exploratory indicators of disproportionate contribution to the observed table, while bias-corrected Cramér’s V was used as an effect-size diagnostic. Counts and row percentages constituted the primary descriptive results, and Monte Carlo *p*-values, adjusted residuals, and Cramér’s V were used to characterize the magnitude and distribution of the observed patterns. All analyses were conducted in R (R Core Team 2021). Online Resource 1 provides the coding matrix and complete analytical diagnostics, including contingency tables, expected counts, adjusted standardized residuals, and the C2 sensitivity analyses; Online Resource 2 provides the R code used to reproduce C1, C2, C2-S1, C2-S2, C3, and C4.

AI-assisted tools were used to support source checking, structured extraction, record organization, and consistency checks. The authors retained full responsibility for coding decisions, analytical choices, interpretation, and final verification.

The table summarizes the main conceptual and analytical dimensions used to characterize the 94 studies. Detailed coding rules, auxiliary fields, and the complete coding matrix are provided in Online Resource 1.

## Results

### General overview of the review

The final synthesis included 94 studies concerned with group selection, multilevel selection, kin selection, inclusive fitness, altruism, and model-based explanations of social evolution. The largest predominant explanatory orientation was Evolutionary Change (33 studies; 35.1%), followed by Adaptationist/KS (27; 28.7%), Hybrid (18; 19.1%), and Unclassifiable/other (16; 17.0%). Thus, a substantial proportion of the reviewed corpus combined both explanatory targets or could not be assigned a predominant orientation within the two main traditions.

The quantitative synthesis examined associations between predominant explanatory orientation and formal tool, decomposition role, and equivalence position, together with the relation between predominant Research Question and MSI/MSII classification. The main exploratory contingency-table diagnostics are summarized in Table 2. Complete contingency tables, row percentages, expected counts, adjusted standardized residuals, and additional diagnostics are provided in Online Resource 1.

**Table 2:** Main contingency-table diagnostics

Analysis	Crossing	N	χ^2^ (df)	Monte Carlo *p*	Bias-corrected Cramér’s V	Expected < 5
C1	Predominant explanatory orientation × predominant formal tool	94	20.666 (12)	0.048	0.177	12/20
C2	Predominant explanatory orientation × primary decomposition role	94	51.583 (12)	< 0.001	0.380	14/20
C2-S1	C2 excluding Unclassifiable/other	78	14.713 (8)	0.055	0.209	11/15
C2-S2	Adaptationist/KS × Evolutionary Change, by primary decomposition role	60	3.574 (3)	0.335	0.094	4/8
C3	Predominant Research Question × MSI/MSII classification	81	0.723 (4)	1.000	0.000	4/9
C4	Predominant explanatory orientation × collapsed equivalence position	68	6.985 (3)	0.070	0.243	4/8

C2-S1 excludes Unclassifiable/other studies; C2-S2 includes only Adaptationist/KS and Evolutionary Change studies. Expected <5 indicates the number of cells with expected counts below five relative to the total number of cells. All contingency analyses are treated as exploratory. Full observed counts, row percentages, expected counts, adjusted standardized residuals, and sensitivity analyses are provided in Online Resource 1; the corresponding R code is provided in Online Resource 2. C4 uses the collapsed equivalence position as a secondary exploratory summary; detailed equivalence positions and equivalence objects are reported descriptively in the Results and Online Resource 1

The table summarizes the exploratory diagnostics for the four planned contingency analyses and the two sensitivity analyses of C2. Counts and row percentages constitute the primary descriptive results; Monte Carlo *p*-values and bias-corrected Cramér’s V are reported as exploratory diagnostics.

### Explanatory orientation and formal tools

The association between predominant explanatory orientation and predominant formal tool was weak and borderline, with substantial sparsity (C1, n = 94; Table 2). No formal tool was exclusive to a single explanatory orientation.

Decompositional/statistical tools were the most frequent category among Adaptationist/KS studies (11/27; 40.7%), Evolutionary Change studies (17/33; 51.5%), and Unclassifiable/other studies (8/16; 50.0%). Dynamic/stochastic tools were particularly frequent among Hybrid studies (11/18; 61.1%), compared with 7/27 Adaptationist/KS studies (25.9%) and 11/33 Evolutionary Change studies (33.3%). Optimization/game-theoretic tools occurred in 4/27 Adaptationist/KS studies (14.8%) and 3/16 Unclassifiable/other studies (18.8%) but were not the predominant formal tool in any Evolutionary Change or Hybrid study. Mixed/multiple and Other/undefined tools occurred across several orientations at lower frequencies. Overall, the distribution indicates some variation in relative tool use while also showing considerable overlap among explanatory orientations.

### Explanatory orientation and decomposition role

The full association between predominant explanatory orientation and primary decomposition role was stronger than the other contingency patterns but was also highly sparse (C2, n = 94; Table 2). The largest disproportionate contribution came from Unclassifiable/other studies classified as Methodological/comparative: 14 of 16 studies in this orientation (87.5%) had this primary decomposition role, with an adjusted standardized residual of 6.053.

The distribution among the other orientations differed substantially from this pattern. Causal-explanatory decomposition was the most frequent role in Adaptationist/KS (15/27; 55.6%), Evolutionary Change (20/33; 60.6%), and Hybrid studies (11/18; 61.1%). Accounting/diagnostic uses were uncommon in all three orientations, occurring in 2/27 Adaptationist/KS studies (7.4%), 1/33 Evolutionary Change study (3.0%), and 2/18 Hybrid studies (11.1%). Methodological/comparative uses occurred in 3/27 Adaptationist/KS studies (11.1%) and 8/33 Evolutionary Change studies (24.2%), but in none of the Hybrid studies. Decomposition was classified as Not used in 7/27 Adaptationist/KS studies (25.9%), 4/33 Evolutionary Change studies (12.1%), and 3/18 Hybrid studies (16.7%).

Because the full C2 pattern could be influenced by the concentration of Methodological/comparative studies within Unclassifiable/other, two sensitivity analyses were examined. After excluding Unclassifiable/other, the association was substantially attenuated (C2-S1; Table 2). When the comparison was restricted further to Adaptationist/KS and Evolutionary Change, the association became very weak (C2-S2; Table 2). Thus, the strong pattern in the full contingency table was not reproduced in the direct comparison between the two principal explanatory orientations.

### MSI/MSII classification and Lloyd’s research questions

The relationship between predominant Research Question and MSI/MSII classification showed no detectable association (C3, n = 81; Table 2).

RQ1, the interactor Research Question, was the predominant RQ in 56 of these studies. Of these, 25 (44.6%) were classified as MSI, 25 (44.6%) as MSII, and 6 (10.7%) as Mixed. The four studies predominantly addressing RQ2, the replicator/reproducer Research Question, were evenly divided between MSI and MSII (2 each). Among the 21 studies predominantly addressing RQ3, the manifestor-of-adaptation Research Question, 9 (42.9%) were classified as MSI, 9 (42.9%) as MSII, and 3 (14.3%) as Mixed. Similar distributions across RQ categories provide no evidence of a direct correspondence between predominant Research Question and MSI/MSII classification in this sample. No study in the full coding was classified as predominantly addressing RQ4, the beneficiary Research Question.

### Equivalence positions across explanatory orientations

Equivalence was substantively discussed in 68 of the 94 studies (72.3%). The proportion was similar among Adaptationist/KS studies (19/27; 70.4%) and Evolutionary Change studies (23/33; 69.7%), lower among Hybrid studies (10/18; 55.6%), and 16/16 Unclassifiable/other studies discussed equivalence. Subsequent distributions of equivalence position and object therefore refer to these 68 studies.

The detailed positions show that the dominant pattern was not a simple opposition between affirmation and rejection of equivalence. Mixed/case-dependent positions were the most frequent category (39/68; 57.4%), followed by formally equivalent, explanatorily distinct (14/68; 20.6%), Equivalent/formally equivalent without explanatory qualification (12/68; 17.6%), and Not equivalent (3/68; 4.4%). Thus, 53 of the 68 studies (77.9%) adopted explicitly qualified positions, either restricting the explanatory consequences of formal equivalence or treating equivalence as dependent on the formulation, assumptions, or case under consideration. Categorical rejection of equivalence was comparatively rare.

The object of equivalence was also usually broader than formal or mathematical equivalence alone. Multiple objects were coded in 51/68 studies (75.0%), whereas 9/68 (13.2%) addressed Formal/mathematical equivalence alone, 6/68 (8.8%) Causal/explanatory equivalence, and 2/68 (2.9%) Predictive/empirical equivalence. Equivalence claims in the reviewed corpus are therefore commonly concerned with more than one relation among alternative formulations, rather than a single question of mathematical translatability.

Detailed positions varied descriptively across explanatory orientations, although the cross-tabulation was too sparse to support an additional omnibus test. Mixed/case-dependent was the modal position in all four orientations. Equivalent/formally equivalent without explanatory qualification positions were relatively more frequent among Adaptationist/KS studies (7/19; 36.8%) than among Evolutionary Change (3/23; 13.0%), Hybrid (1/10; 10.0%), or Unclassifiable/other studies (1/16; 6.3%). Formally equivalent, explanatorily distinct positions occurred in all orientations, while the three not equivalent cases were confined to Adaptationist/KS and Evolutionary Change studies.

The relation between equivalence object and detailed position further illustrates the qualified character of these claims. Thirteen of the 14 studies classified as formally equivalent, explanatorily distinct and 31 of the 39 Mixed/case-dependent studies addressed multiple objects of equivalence. By contrast, among the nine studies concerned exclusively with Formal/mathematical equivalence, five were classified as Equivalent/formally equivalent without explanatory qualification. Because this cross-tabulation also contained highly sparse margins, these patterns were treated descriptively rather than as a basis for an additional inferential test.

For comparison with the other contingency analyses, C4 retained a collapsed distinction between Affirms equivalence and Restricts/rejects equivalence. Under this aggregation, 12 studies affirmed equivalence and 56 restricted or rejected it. The association with predominant explanatory orientation showed a moderate descriptive tendency but no clear departure from independence (C4, n = 68; Table 2). This collapsed result should be interpreted considering the detailed distribution above: 53 of the 56 studies grouped as Restricts/rejects equivalence occupied qualified categories, whereas only three were classified as not equivalent.

## Discussion

### Main interpretation: explanatory function, not formal equivalence alone

This review shows that, within the model-centered literature examined here, the debate on group and multilevel selection cannot be reduced to the question of whether group-selection and kin-selection models are formally equivalent. Formal equivalence remains an important part of the controversy, but the reviewed literature also varies in the Research Questions being addressed, the explanatory targets emphasized, the functions assigned to formal representations, and the causal interpretations attached to them (Okasha 2006, 2016; Jeler 2020; Lehtonen 2020; Lloyd 1994, 2024; Suárez and Lloyd 2023). These dimensions overlap, but the results indicate that they should not be treated as interchangeable.

The distribution of predominant explanatory orientations illustrates this point. Although Adaptationist/KS and Evolutionary Change orientations were both recurrent in the reviewed corpus, 34 of the 94 studies (36.2%) were classified as Hybrid or Unclassifiable/other. Moreover, formal-tool use overlapped substantially across orientations, and the direct comparison between Adaptationist/KS and Evolutionary Change studies provided little support for the idea that they are distinguished by systematically different roles assigned to decomposition. The distinction between these traditions is therefore most useful as a contrast in predominant explanatory emphasis, rather than as a division between mutually exclusive methodological schools.

This more differentiated picture also changes how persistent disagreement should be interpreted. A study’s predominant Research Question, MSI/MSII classification, formal tool, decomposition role, causal interpretation, and position on equivalence capture related but conceptually distinct aspects of its explanatory structure. The comparisons examined here indicate incomplete correspondence among several of these dimensions, rather than allowing them to be treated as interchangeable. Agreement along one of these dimensions does not imply agreement on the others. Conversely, studies may employ similar mathematical tools or analyze similar evolutionary processes while assigning them different explanatory significance. The main conceptual distinctions underlying this interpretation are summarized in Table 3.

**Table 3:** Conceptually distinct dimensions structuring the debate on group and multilevel selection

Conceptual axis	Main distinction	What it clarifies
Explanatory orientation	Adaptationist/KS; Evolutionary Change; Hybrid; Unclassifiable/other	Characterizes the predominant explanatory emphasis of a study, particularly whether adaptive explanation, evolutionary change, both, or neither provides its principal explanatory payoff
Research questions	Interactor; replicator/reproducer; manifestor-of-adaptation; beneficiary	Identifies which evolutionary role or explanatory question is being investigated
MSI/MSII classification	MSI: individual trait change in group-structured populations; MSII: differential persistence or reproduction of collectives	Distinguishes different ways in which multilevel evolutionary change and fitness can be represented
Formal representation	Price partitions; contextual analysis; inclusive-fitness formulations; quantitative-genetic and dynamic models; optimization; games	Identifies how evolutionary relationships, fitness effects, or change are represented mathematically
Decomposition role	Accounting/diagnostic; causal-explanatory; methodological/comparative; mixed; not used	Distinguishes the analytical or explanatory function assigned to a decomposition
Causal interpretation of decomposition	Causally agnostic; causally interpreted; conditional/underdetermined; causal interpretation rejected	Characterizes how a decomposition is related to the causal structure attributed to the evolutionary process
Equivalence	Formal/mathematical; predictive/empirical; causal/explanatory; multiple	Clarifies what is claimed to be equivalent and whether that equivalence is treated as unrestricted, explanatorily limited, case-dependent, or absent
Adaptation	Selection-product adaptation; engineering adaptation	Distinguishes being a product of selection from the stronger attribution of design-like functional organization

“Engineering adaptation” refers to adaptation in the stronger functional sense of design-like organization, whereas “selection-product adaptation” refers more broadly to a trait or state resulting from selection. The dimensions summarized here are conceptually distinct and should not be treated as interchangeable; their empirical correspondence was assessed only in the specific comparisons reported in the Results

The table summarizes the main conceptual axes used in this review to interpret persistent disagreement in the reviewed articles. These distinctions clarify why formal equivalence between models does not necessarily imply equivalence in research questions, causal interpretation, or explanatory function.

### Formal equivalence and its explanatory consequences

Formal equivalence remains central to the debate because alternative formulations of social evolution can often be translated into one another under specified assumptions. Such translations are informative: they can reveal common mathematical structure, identify equivalent conditions for evolutionary change, or show that apparently competing formulations yield the same result within a particular domain (Queller 1992; Lehmann et al. 2007; Gardner et al. 2011; Marshall 2011; Lehtonen 2020). However, the significance of a translation depends on what is claimed to be equivalent. Formal or mathematical equivalence concerns relations between formal representations; predictive or empirical equivalence concerns their observable consequences; causal or explanatory equivalence concerns whether they represent the same process in the same explanatory terms (Okasha 2016; Jeler 2020; Lehtonen 2020; Thies and Watson 2021). These relations may coincide in particular cases, but one does not follow automatically from another.

The detailed results show that the reviewed studies were rarely divided between unrestricted affirmation and categorical rejection of equivalence. Among the 68 studies that substantively discussed equivalence, Mixed/case-dependent positions were most frequent, followed by studies that accepted formal equivalence while maintaining an explanatory distinction. Together, these qualified positions accounted for 53 of the 68 studies, whereas only three were classified as Not equivalent. Moreover, three quarters of the substantive discussions involved multiple objects of equivalence rather than formal or mathematical equivalence alone. The main pattern in this model-centered corpus is therefore one of conditional, qualified, and multidimensional equivalence rather than a binary dispute over whether equivalence exists.

This pattern also clarifies what is at stake when two formulations are described as equivalent. Equivalence can be established at different levels of comparison: between algebraic expressions, between models under a specified mapping and shared assumptions, or between broader model interpretations that include claims about causal structure and explanatory relevance. Demonstrating equivalence at the first level need not establish equivalence at the others. Conversely, differences in interpretation do not by themselves show that the underlying formal representations are mathematically incompatible. The relevant question is therefore not simply whether a translation can be constructed, but what the translation preserves, under which assumptions it holds, and which explanatory claims remain invariant under it.

The variation across explanatory orientations is consistent with this more qualified interpretation. Mixed/case-dependent positions were modal in all four orientations, although unqualified equivalence was relatively more frequent among Adaptationist/KS studies. This difference should not be read as a division between one orientation that accepts equivalence and another that rejects it: restrictive or qualified positions were common throughout the corpus, and the detailed crossing was too sparse to support a stronger inferential claim. Van Veelen’s analyses provide a useful example of why the scope of equivalence matters, showing how conclusions about equivalence can depend on the particular formulations, assumptions, and domains being compared rather than supporting an unrestricted equivalence between approaches (van Veelen 2005, 2020).

The results therefore support a more specific claim than either universal equivalence or general explanatory incompatibility. Formal equivalence can establish genuine relations between evolutionary models, but the explanatory consequences of those relations depend on the object of comparison, the assumptions under which the equivalence is obtained, and the biological question being addressed. In the reviewed corpus, the predominance of case-dependent and explicitly qualified positions indicates that the controversy is often not over whether some form of equivalence can be demonstrated, but over its scope and what follows from it for causal interpretation and explanatory function.

### Decomposition, causal interpretation, and multilevel partitions

The results do not support treating decomposition role as a defining contrast between the Adaptationist/KS and Evolutionary Change orientations. Although the full contingency table showed a pronounced association, this pattern was strongly influenced by the concentration of Methodological/comparative uses among Unclassifiable/other studies. Once these studies were excluded, the association weakened substantially, and the direct comparison between Adaptationist/KS and Evolutionary Change showed very similar frequencies of Causal-explanatory decomposition. Decomposition role therefore appears better understood as an analytical dimension that cuts across explanatory orientations than as a marker distinguishing two theoretical traditions.

This distinction is important because the use of a decomposition and the causal interpretation assigned to it are separate issues. A decomposition may partition evolutionary change, quantify covariance components, compare alternative formulations, or contribute to a causal explanation. Even when authors use a partition causally, however, the existence of mathematically identifiable components does not by itself establish that those components correspond to distinct causal processes. The causal significance of a decomposition depends on how its terms relate to the process generating the associations represented in the model. This is why the present synthesis distinguishes the primary role assigned to decomposition from the causal status attributed to that decomposition.

The Price equation illustrates this point particularly clearly. As a general identity, it can support several kinds of analysis, including comparisons among kin-selection and multilevel formulations and partitions of evolutionary change across hierarchical structure (Price 1970, 1972; Frank 2012; Lehtonen 2020). Its generality does not make it either inherently non-causal or causal. In cases where differences between groups appropriately track higher-level selective effects, a multilevel Price partition may represent the relevant causal organization of selection. In other cases, individual-level selection can also contribute to fitness differences across groups, so that the between-group component cannot be identified wholesale with higher-level selection (Jeler 2020; Shelton and Michod 2020). Contextual analysis may then separate individual and contextual effects more appropriately, although its causal adequacy likewise depends on the structure of the particular system rather than on the method alone (Heisler and Damuth 1987; Okasha 2016; Jeler 2020; Thies and Watson 2021). Moreover, contextual-analysis partitions can themselves be closely related to formulations used in kin-selection analysis, further weakening any simple association between a formal method and a theoretical orientation (Okasha 2016).

The reviewed literature also provides concrete examples of why explanatory orientation should not be inferred from methodological vocabulary alone. Muir (2005) and Bijma et al. (2007) both address social interactions in applied breeding contexts and employ closely related quantitative-genetic reasoning, yet their principal explanatory payoffs differ. Muir (2005) focuses on how competitive effects can be incorporated into breeding programmes to improve the outcome of selection, whereas Bijma et al. (2007) develops the inheritance and response-to-selection consequences of direct and associative genetic effects. Their methodological proximity therefore coexists with different predominant explanatory emphases, illustrating more generally why formal representation and explanatory orientation need to be kept analytically distinct.

Causal interpretation also depends on how the system represented by a model is delimited. Population structure, dispersal, social interactions, and other contextual features may enter one model as fixed background conditions but become variables generated or modified by evolutionary dynamics in another. The distinction matters because a factor treated as context need not be causally external to the process under explanation. When population structure itself changes together with social behaviour, for example, separating a focal trait from its evolutionary environment becomes partly dependent on how the model represents their reciprocal dynamics.

Powers et al. (2011) provide an example within the reviewed literature: population structure is not treated solely as an externally imposed setting for the evolution of cooperation but participates in the evolutionary dynamics being analysed. Such cases extend the point made by multilevel partitions. A decomposition can correctly characterize evolutionary change under a particular specification while its causal interpretation still depends on which variables are treated as focal, contextual, fixed, or endogenous. This does not make model boundaries arbitrary, but it makes their biological justification part of the explanatory problem.

The broader implication is that neither the name of a partition nor the explanatory orientation in which it appears determines what causal conclusions can be drawn from it. Between-group and within-group terms, contextual effects, inclusive-fitness components, and related decompositions acquire explanatory significance through their relation to model assumptions, the boundaries of the system being represented, and the evolutionary process the model is intended to capture. The relevant distinction is therefore not between inherently causal and merely accounting formalisms, but between uses for which the causal interpretation is warranted by the specified biological process and uses for which the decomposition remains descriptive, comparative, or underdetermined.

### Research questions, MSI/MSII, and the attribution of adaptation

The absence of a detectable association between predominant Research Question and MSI/MSII classification is consistent with this separation. MSI and MSII characterize different organizations of multilevel evolutionary change, whereas the Research Questions characterize the explanatory role being investigated. A study concerned primarily with the interactor Research Question can therefore be formulated in either MSI or MSII terms, just as a study concerned with adaptation need not map uniquely onto one of these forms of multilevel selection. The similar distribution of MSI and MSII across the predominant Research Questions in the reviewed studies provides no basis for treating correspondences such as RQ1–MSI or RQ3–MSII as general equivalences.

This lack of direct correspondence also helps clarify the status of the MSI/MSII distinction itself. The distinction remains useful for separating cases in which evolutionary change is tracked through individual traits in group-structured populations from cases in which collectives themselves differ in persistence or reproduction. At the same time, genetical formulations do not always map neatly onto this dichotomy, and the distinction need not imply two universally separable evolutionary processes (Gardner 2015; Bourrat 2023). Its usefulness therefore lies in identifying differences in how multilevel change and fitness are represented, rather than in providing a complete classification of the explanatory questions addressed by a model.

More generally, Research Questions, MSI/MSII classification, and adaptation claims should be treated as related but non-redundant dimensions. A model may identify a group-level interactor without attributing engineering adaptation to the group; it may represent multilevel change without resolving the beneficiary question; and it may explain the evolution of an adaptive trait without requiring that the level at which selection is partitioned coincide with the level at which adaptation is attributed. Keeping these dimensions distinct helps specify more precisely what a model establishes and what remains an additional explanatory claim.

### Limitations

It is important to point out certain limitations of the present study. First, the search was conducted only in *Web of Science* and *Scopus* and was not updated after 4 July 2025. Books, book chapters, older theoretical works, studies not indexed in these databases, and literature published after the search date may therefore be underrepresented. This limitation is particularly relevant to a debate with a long conceptual history and important contributions in monographs and edited volumes. The results should consequently be interpreted with reference to the corpus retrieved by the specified search rather than as an exhaustive representation of the entire literature on social evolution.

Second, the search strategy was deliberately model-centered. It was restricted to title and keyword fields and included the term “model*”, while eligibility required explicit engagement with group selection or multilevel selection. This design was appropriate for the review question but does not provide a symmetrical survey of all work on kin selection, inclusive fitness, the Price equation, or social evolution more broadly. Studies using these approaches without explicitly framing their contribution in relation to group or multilevel selection may be underrepresented. The observed frequencies of explanatory orientations and equivalence positions therefore characterize the model-centered literature retrieved by this search, not the full distribution of positions across evolutionary theory.

Third, the coding scheme involved interpretive decisions. Predominant explanatory orientation was reconstructed from independently coded adaptive and evolutionary-change targets, while predominant Research Question, decomposition role, causal interpretation, and equivalence position likewise required judgments about the principal argumentative and explanatory content of each study. All classifications were made by a single coder, and no independent estimate of inter-coder agreement was obtained. Online Resource 1 provides the coding rules, study-level evidence, confidence assessments, analytical decisions, and complete coding matrix to make these judgments auditable. Nevertheless, the resulting classifications should be treated as explicit analytical reconstructions of the studies rather than as directly observed or error-free properties.

Fourth, the quantitative unit of analysis was the study rather than each individual model, derivation, or model comparison contained within a study. Predominant classifications summarize the main explanatory or argumentative structure of each paper, but some studies compare several formulations, use more than one formal tool, or adopt different equivalence positions under different assumptions. The synthesis is therefore suited to identifying study-level patterns in the reviewed literature, but frequencies of coded studies should not be interpreted automatically as frequencies or universal properties of the underlying models themselves.

Fifth, several contingency tables contained sparse expected counts, and some categories were represented by few studies. This limits the precision of omnibus comparisons and the interpretive weight that can be assigned to individual cells. Counts and row percentages were therefore treated as the primary results, while Monte Carlo p-values, bias-corrected Cramér’s V, expected counts, and adjusted standardized residuals were used as exploratory diagnostics. The strong association in the complete C2 table also depended substantially on the concentration of Methodological/comparative studies within the Unclassifiable/other orientation; the sensitivity analyses were therefore necessary for evaluating the comparison among the principal explanatory orientations. The reported crossings should be understood as specific tests of correspondence among selected dimensions, not as evidence that the full set of coding dimensions is statistically independent.

Finally, the review reconstructs how studies formulate causal and explanatory claims; it does not independently establish which decomposition or formal representation provides the correct causal account of any particular biological system. The causal adequacy of Price partitions, contextual analyses, inclusive-fitness formulations, or other representations depends on model assumptions, the generating process, omitted pathways, and the delimitation of focal variables and contextual conditions. Similarly, identifying selection or an interactor at a particular level does not by itself establish engineering adaptation at that level. The conceptual distinctions developed here are therefore intended to clarify the claims made by the reviewed literature and the relations among them, rather than to supply a universal causal criterion for multilevel selection.

### Implications for the group selection debate

The results have several implications for interpreting the group-selection debate represented in this model-centered corpus. First, the distinction between Adaptationist/KS and Evolutionary Change remains useful as a contrast in predominant explanatory emphasis, but it should not be understood as a division between methodologically closed schools. Formal tools overlapped substantially across orientations, and the apparent association between explanatory orientation and decomposition role weakened markedly once method-focused Unclassifiable/other studies were excluded. The explanatory orientations therefore identify recurrent study-level emphases rather than fixed packages of methods, Research Questions, or causal commitments.

Second, the detailed equivalence results shift the relevant contrast away from a simple opposition between acceptance and rejection. Among studies that substantively discussed equivalence, case-dependent positions and explicit acceptance of formal equivalence with explanatory qualification predominated, whereas categorical non-equivalence was rare. Moreover, most of these studies addressed multiple objects of equivalence rather than formal or mathematical equivalence alone. The central issue is therefore often not whether some translation between formulations exists, but what is equivalent, under which assumptions, and what explanatory consequences follow from that equivalence. A formal mapping can establish a genuine relation between representations without establishing that the corresponding models make the same causal commitments, answer the same Research Question, or support the same attribution of adaptation.

Third, the results caution against using one classificatory dimension as a proxy for another. Predominant Research Question showed no detectable correspondence with MSI/MSII classification in the analyzed subset, while formal tools and decomposition roles likewise failed to define the principal explanatory orientations in a simple way. These findings do not establish statistical independence among the full set of dimensions examined in the review. They instead show that the specific dimensions compared here were incompletely aligned and cannot be treated as interchangeable descriptions of the same theoretical distinction. Research Questions, MSI/MSII classifications, formal representations, decomposition roles, causal interpretations, and equivalence claims therefore need to be specified according to the particular inference being made.

This has a corresponding implication for causal interpretation. Neither the name of a formalism nor the position of a term within a decomposition determines its causal status. Price-equation partitions, contextual analyses, inclusive-fitness formulations, and related representations can contribute to causal explanation when their assumptions and terms appropriately represent the generating process, but the same mathematical objects can also be used descriptively or comparatively. Likewise, identifying an interactor or a component of selection at a particular level does not by itself establish engineering adaptation at that level. Evaluating a model therefore requires distinguishing the mathematical relation it represents from the biological and explanatory claims attached to that representation.

Taken together, the review supports a pluralist interpretation of multilevel explanation without implying unrestricted equivalence among alternative formulations. Different representations may legitimately illuminate different aspects of an evolutionary process, but their adequacy depends on the question being asked, the assumptions under which they are derived, and the biological process they are intended to represent. Formal equivalence can establish translatability under specified conditions; it does not, by itself, determine whether two formulations preserve the same causal structure, explanatory function, or attribution of adaptation.

The central conclusion is therefore narrower than a claim that the major traditions of social evolution constitute independent theoretical systems. In the 94 model-centered studies retrieved by this review, explanatory orientations were heterogeneous, formal and analytical practices overlapped, Research Questions and MSI/MSII classifications did not correspond directly, and equivalence was most often treated as conditional, qualified, or multidimensional. These patterns indicate that the relevant dimensions of the debate are conceptually distinct and incompletely aligned in the comparisons examined. Recognizing those distinctions clarifies both what formal equivalence can establish and why disagreement can persist after formal translatability has been demonstrated.

## Supplementary Information

Below is the link to the electronic supplementary material.

Supplementary Material 1

Supplementary Material 2

## Data Availability

The review coding matrix and contingency-table diagnostics supporting the findings of this study are available as Online Resource 1.

## Code availability

The R script used for the exploratory contingency-table diagnostics is available as Online Resource 2.
